# Vaccinal Syphilis

**Published:** 1869-12

**Authors:** 


IMPERIAL Academy of Medicine. Session of September 14, 1869.

M. Chassaignac read the following paper upon the subject of Vaccinal Syphilis:

Without taking any direct part in the discussion, I desire to present a document of a character I believe to dissipate the doubts expressed by several of our colleagues.

The first example of vaccinal syphilis which has been demonstrated in the hospitals of Paris dates from 1863. It is, consequently, much anterior to the majority of the evidence adduced in the discussion.

This fact, regarded as demonstrating the real existence of vaccinal syphilis, or syphilis by vaccination, is of decisive precision and authenticity. It had for witnesses the medical staff of one of the large hospitals of Paris; and upon two successive occasions during the evolution of vaccinal-syphilitic-symptoms, a society perfectly competent upon questions of this character.

A child, aged two years, which had been nursed by its mother, and whose father and mother were both free from every syphilitic affection, was brought into my department, at Lariboisière, the 18th of August, 1863. This child had been vaccinated at the mayoralty of Montmartre, seven weeks before, the 27th of June, 1863. Upon the third day the first vaccinal eruption developed itself; the pustules suppurated toward the 9th of June (July?). They dessicated, the crusts fell the 15th day, and the cicatrices appeared definite.

Some days afterwards, three of the vaccinal cicatrices were replaced by ulcerations which suppurated, enlarged, assumed the dimensions of a 50-centime piece, became covered with a crust thick at the edges, thin in the centre. These ulcerations were indolent and rested upon an indurated base. The axillary ganglia were engorged, as were likewise the cervical ganglia. Upon the right ear a copper-colored papule appeared, covered with little grayish scales of entirely characteristic appearance. Upon the chest, the abdomen, and the back, appeared an eruption presenting a slight relief of a red color, coppery at certain points, especially upon the upper portion of the chest.

The surgical society, through its members most competent in such matters, recognized without hesitation, and positively affirmed the existence of vaccinal syphilis.

Lastly, in order to shun every pretext for dispute, the child was submitted to no treatment up to the second presentation, which occurred eight days from date. At this second session the affirmations of my colleagues were yet more decisive and more circumstantial. The coppery patches which, eight days before, commenced to appear, were upon the 27th of September altogether characteristic. The English physicians caused a colored drawing of the little syphilitic to be taken. Doctor Druitt presented to the obstetrical society of London the drawings taken on the 19th of September; that is to say, twelve weeks after the vaccination.

M. Chassaignac exhibited these drawings to his colleagues.—*Gazette des Hopitaux, Paris.*

In the course of his remarks upon animal vaccination, made before the Academy of Medicine, Paris, September 24, 1869, M. Jules Guérin asserted his belief that Jennerian (human) vaccine, in general, had not undergone degeneration, whilst it undoubtedly had done so, in exceptional cases, temporarily, and from various causes. He quotes, in support of his opinion, M. le docteur d'Aquino Fonseca, of Pernambuco (Brazil), Commissioner-General of Vaccine, who, during a series of years, produced exactly similar results from the repeated use of the same virus, transmitted from subject to subject. M. Caradec, physician to the hospital at Brest, and M. Brochard, of Nogent le Rotru, who, after transmitting the virus through "*thousands*" of cases, found it unimpaired in activity. Also M. Bonafont, who, having originally introduced vaccine into Algeria, in 1834–5, by means of two crusts forwarded to him by the Academy, at Paris, with which he vaccinated four infants, and the virus from this, the sole source of vaccine in Algeria, has been transmitted to the present time unimpaired.

With regard to vaccinal syphilis, which he alleges to be the principal pretext for the substitution of animal for human vaccine, he indorses the assertion of M. Blot, "that neither M. Depaul, nor any one else, has advanced a single clear and authentic fact capable of demonstrating that vaccine virus alone has ever inoculated syphilis."

And, further, he asserts that "the experiments of M. Delzenne prove positively that the inoculation of vaccine virus taken from a *syphilitic vaccinifere* is absolutely incapable of producing vaccinal syphilis."

Finally, "I believe I have established, on the one hand, the impossibility of the simultaneous existence of the vaccine pustule and the infecting chancre, and, on the other, the necessary succession and separation of these two transitory organisms in order to produce the virus which belongs to them."

The Surgery of the Italian Campaign of 1866. Clinical observations by Doctor Rodolphi.

After a relation of the prominent surgical incidents observed during the last campaign for Italian independence, the author traces conclusions which he hopes "will not be useless to those who may be called in the future to aid other victims of war."

1st. Permanent and absolute immobility of a limb wounded in one of its articulations suspends pain, and prevents nearly always the necessity of amputation.

2nd. The apparata of Bonnet, of iron or copper wire, being elastic, and not rigid, are not appropriate to favor the healing of wounds of articulations; applied to fractures, they permit frequent and painful contractions in the wounded limbs, because they transmit the oscillations of the bed, or perhaps because they develop electric currents when the wounds are covered with emollient cataplasmata.

3rd. Amputations of limbs should be avoided as much as possible, and should be resorted to only when there is clear evidence that there is remaining no probability of cure by means of conservative surgery.

4th. Flap amputations resulted in the most prompt cures. Teale's method for amputation of the thigh should be preferred in cases of free election, because it secures the impossibility of protrusion of the stump, a free issue for the pus, and a cicatrix of favorable form. The patella-flap of Gritti may afford the best results.

5th. The internal administration of sulphites is not the most certain means to establish the truth of the doctrine of ferments of Professor Polli. The development of sulphites in the intestinal tube is the best means to attain, probably, incontestible results.

6th. The only means to arrest hæmorrhages of the face, produced by gun-shot wounds, is the application of ice with pressure. The per-chloride of iron sometimes determines severe syncope; the other hæmostatics, astringent and caustic, are of difficult application; and the actual cautery, especially when the wound communicates with the baccal cavity, can not reach the openings through which the blood escapes.

7th. Drainage has demonstrated, once more, its practical utility in purulent infiltrations, and in vast collections of pus.

8th. Nélaton's probe may induce grave errors with regard to the locality in which a projectile may be found. With a canula, which a porcelain button secures from every contact, nothing better is required.

The electric sound of Favre, concealed in a protecting tube of gutta-percha, may supply a valuable assistant in the discovery of a ball buried in the tissues, especially by adapting to it the modifications which we have proposed. We have not experimented with the electric sound.

9th. Ball-forceps, with toothed blades, are, so to speak, abandoned; those which are better adapted to the new system of conical balls, are those already used by American surgeons, and especially those of Ferguson, modified by us.—*Gazetta Medica Italiana di Lombardia.*

A PRISONER at Pentonville prison (England) was seized suddenly with vomiting of blood, and at the end of a few hours died from these repeated hæmorrhages. At the autopsy a counterfeit half-crown piece was discovered lodged in a pouch formed in the æsophagus, and which had occasioned ulceration and perforation of the aorta. The prisoner had been engaged in passing counterfeit money, and one day, in order to avoid detection, had swallowed this half-crown. This incident occurred ten or eleven months before his death. A remarkable feature in the case was the entire absence of all difficulty in deglutition, or of any other symptom which could indicate the presence of a foreign body in the æsophagus.—*British Med. Jour. Gazette Medicale.*

